# Evaluation of acid-labile *S*-protecting groups to prevent Cys racemization in Fmoc solid-phase peptide synthesis

**DOI:** 10.1002/psc.2585

**Published:** 2013-11-28

**Authors:** Hajime Hibino, Yasuyoshi Miki, Yuji Nishiuchi

**Affiliations:** a SAITO Research Center, Peptide Institute, Inc.Ibaraki, Osaka, 567-0085, Japan; b School of Pharmaceutical Sciences, Kinki UniversityHigashi-Osaka, Osaka, 577-8502, Japan; c Graduate School of Science, Osaka UniversityToyonaka, Osaka, 560-0043, Japan

**Keywords:** cysteine, 4,4′-dimethoxydiphenylmethyl (Ddm), 4-methoxybenzyloxymethyl (MBom), microwave (MW)-assisted solid phase peptide synthesis (SPPS), phosphonium reagent, protecting group, racemization, uronium reagent

## Abstract

Phosphonium and uronium salt-based reagents enable efficient and effective coupling reactions and are indispensable in peptide chemistry, especially in machine-assisted SPPS. However, after the activating and coupling steps with these reagents in the presence of tertiary amines, Fmoc derivatives of Cys are known to be considerably racemized during their incorporation. To avoid this side reaction, a coupling method mediated by phosphonium/uronium reagents with a weaker base, such as 2,4,6-trimethylpyridine, than the ordinarily used DIEA or that by carbodiimide has been recommended. However, these methods are appreciably inferior to the standard protocol applied for SPPS, that is, a 1 min preactivation procedure of coupling with phosphonium or uronium reagents/DIEA in DMF, in terms of coupling efficiency, and also the former method cannot reduce racemization of Cys(Trt) to an acceptable level (<1.0%) even when the preactivation procedure is omitted. Here, the 4,4′-dimethoxydiphenylmethyl and 4-methoxybenzyloxymethyl groups were demonstrated to be acid-labile *S*-protecting groups that can suppress racemization of Cys to an acceptable level (<1.0%) when the respective Fmoc derivatives are incorporated via the standard SPPS protocol of phosphonium or uronium reagents with the aid of DIEA in DMF. Furthermore, these protecting groups significantly reduced the rate of racemization compared to the Trt group even in the case of microwave-assisted SPPS performed at a high temperature. © 2013 The Authors. *European Peptide Society* published by John Wiley & Sons, Ltd.

## Introduction

In peptide chemistry, phosphonium and uronium salt-based coupling reagents, such as PyBOP and HBTU, have been widely employed for highly efficient coupling, leading to rapid coupling and elimination of side products, such as deletion and truncated peptides. These reagents are especially useful for the coupling of sterically hindered amino acids and in machine-assisted SPPS involving stepwise peptide-chain elongation on a solid support 1. Upon the activating and coupling steps with these reagents, however, Fmoc derivatives of Cys and His with their side-chain functionalities protected by the ordinarily used protecting groups, that is, *S*-trityl (Trt)/*S*-Acm and *N^τ^*-Trt groups, are known to be accompanied by a considerable rate of racemization 2,3. Regioselective protection of the *π*-nitrogen of the imidazole ring enables avoidance of racemization during incorporation of the His derivatives, because the unprotected *π*-nitrogen is involved in promoting racemization pathways 4. Therefore, substitution of the *N^π^-t*-Bum 5 or *N^π^*-4-methoxybenzyloxymethyl (MBom) group 6 for the *N^τ^*-Trt group on Fmoc–His offers a solution for eliminating racemization even in the case of microwave (MW)-assisted SPPS performed at a high temperature 7.

On the other hand, the considerable base-catalyzed racemization of the Cys residues always occurs at the activating and coupling steps with phosphonium or uronium reagents where a tertiary amine must be added in order to form the anion of the carboxyl component 8. Thus, racemization of Cys cannot be inherently prevented during its incorporation if the combination of phosphonium/uronium reagents and tertiary amines is employed in the coupling reactions. Disulfide linkages in naturally occurring peptides and proteins play an important role in the formation and stabilization of distinct three-dimensional structures that are closely involved in physiological responses and processes. For the synthesis of such Cys-rich molecules, native chemical ligation involving the coupling of a peptide thioester and a cysteinyl peptide is one of the most advantageous approaches 9. Racemization with Cys arising during its incorporation onto the growing peptide chain may hamper purification of the products including those obtained after the native chemical ligation and disulfide formation reactions. In general, a diastereoisomer with racemization somewhere in its sequence tends to be difficult to separate from the intact peptide as its chain length increases. Therefore, it is important to exclude the risk of racemization with incorporation of Cys during the course of the chain assembly 10. In the present study, we reviewed the phosphonium/uronium reagent-mediated coupling conditions including the choice of the side-chain protecting groups for Cys in order to reduce Cys racemization during its incorporation to an acceptable level (<1.0%).

## Materials and Methods

All reagents and solvents were obtained from the Peptide Institute, Inc. (Osaka, Japan), Wako Chemical (Osaka, Japan), Tokyo Chemical Industry (Tokyo, Japan), and Watanabe Chemical Industries (Hiroshima, Japan). Analytical HPLC was performed on a Shimadzu liquid chromatograph model LC-10AT (Kyoto, Japan) with a DAISO-PAK SP-120-5-ODS-BIO (4.6 × 150 mm) using a flow rate of 1 ml/min and the following solvent systems: 0.1% TFA in H_2_O (A) and 0.1% TFA in MeCN (B). Purities are based upon area percent of the peaks detected at 220 nm. Molecular weights were measured with an ESI-MS (HP1100 LC/MSD, Agilent Technologies, Santa Clara, CA, USA).

## Examination of Cys racemization during synthesis of the model peptide, H–Gly–Cys–Phe–NH_2_

**Figure 1:**
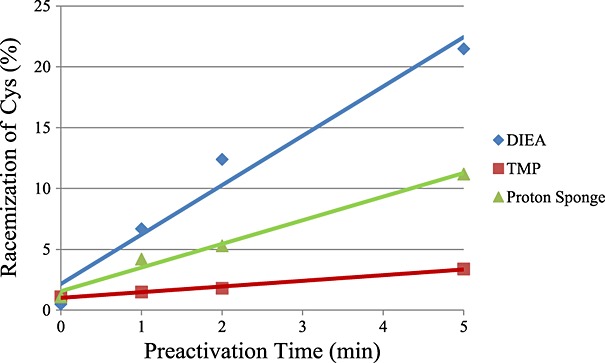
Effect of preactivation time on racemization of Cys. The rate of Cys racemization was determined by synthesizing H–Gly–Cys–Phe–NH_2_ and defined as (H–Gly–d-Cys–Phe–NH_2_)/(H–Gly–l-Cys–Phe–NH_2_) × 100.

Starting with Fmoc–NH–SAL–PEG resin (0.42 g, 0.24 mmol/g), manual peptide chain assembly was carried out. Fmoc–Cys–OH derivatives with the indicated side-chain protection were incorporated by 0–5 min preactivation procedure of coupling with Fmoc–Cys–OH derivative/1-[*bis*(dimethylamino)methylene]-5-chloro-1*H*-benzotriazolium 3-oxide hexafluorophosphate (HCTU)/6-Cl-HOBt/base (4/4/4/8 equiv with respect to the peptide resin; the concentration of active species, 0.2 M; coupling time, 30 min) in DMF or DCM/DMF (v/v, 1/1) as indicated in Figure 1 and Tables 1, 3, and 4. MW heating was performed in a 25 ml polypropylene open vessel placed in the MW cavity of a 300 W single-mode manual MW peptide synthesizer (CEM, Discover SPS) set at 50 or 80 °C; power pulsing sequences of 30 W were used for Cys coupling steps (5 min). Fmoc deprotection was carried out with 20% piperidine in DMF (2 × 7 min), followed by washing with DMF (5 × 2 min). The final release of peptides was achieved with TFA/triisopropylsilane (TIS)/H_2_O (95/5/5) at rt for 1 h. The crude peptides were directly analyzed by HPLC and ESI-MS analyses. HPLC conditions: linear gradient of solvent B in solvent A, 1–60% over 25 min, detection at 220 nm.

**Table 1:** Racemization of Cys during synthesis of the model peptide, H–Gly–Cys–Phe–NH_2_, as a function of the coupling conditions and the Cys protecting group

Coupling conditions^a^				Racemization (%)^b^	
Reagent (4 eq)	Additive (4 eq)	Base (8 eq)	Solvent	Cys(Trt)	Cys(Acm)
HCTU	6-Cl-HOBt	DIEA	DMF	8.0	2.2
HCTU	6-Cl-HOBt	PS	DMF	2.0	0.6
HCTU	6-Cl-HOBt	DBU	DMF	2.1	0.7
HCTU	6-Cl-HOBt	TMP	DMF	1.3	0.3
HCTU	6-Cl-HOBt	DIEA	DCM/DMF (v/v, 1/1)	3.3	1.0
HCTU	6-Cl-HOBt	PS	DCM/DMF (v/v, 1/1)	1.2	0.3
HCTU	6-Cl-HOBt	DBU	DCM/DMF (v/v, 1/1)	1.5	0.4
HCTU	6-Cl-HOBt	TMP	DCM/DMF (v/v, 1/1)	0.6	0.1
DIC	HOBt	—	DMF	0.1	< 0.1
EDC · HCl	HOBt	—	DMF	0.9	0.2

a The HCTU-mediated reactions were performed using a 1 min preactivation procedure of coupling with Fmoc amino acid/HCTU/6-Cl-HOBt (4/4/4 equiv) in the presence of the bases (8 equiv) listed in Table 1. The carbodiimide-mediated reactions were performed by a 5 min preactivation procedure of coupling with Fmoc amino acid/DIC or EDC · HCl/HOBt (4/4/4 equiv). The coupling time was 30 min and concentrations of the active species were 0.20 M.

b Defined as (H–Gly–d-Cys–Phe–NH_2_)/(H–Gly–l-Cys–Phe–NH_2_) × 100.

## Percentages of H–Tyr–Aib–Aib–Phe–Leu–NH_2_ and related deletion peptides obtained in solid-phase assembly

The peptide chain was elongated onto an H–Aib–Phe–Leu Rink amide resin (0.42 g, 0.24 mmol/g) in DMF. Manual peptide chain assembly was carried out using a 1 min preactivation procedure of coupling with Fmoc amino acid/HCTU/6-Cl-HOBt/base (4/4/4/8 equiv with respect to the peptide resin; the concentration of active species, 0.2 M; coupling time, 30 min) in DMF as indicated in Table 2. The DIC-mediated couplings were performed by a 5 min preactivation procedure with DIC/HOBt (4/4 equiv). Fmoc deprotection was carried out with 20% piperidine in DMF (2 × 7 min), followed by washing with DMF (5 × 2 min). The final release of peptides was achieved with TFA/TIS/H_2_O (95/5/5) at rt for 1 h. The crude peptides were directly analyzed by HPLC and ESI-MS analyses. HPLC conditions: linear gradient of solvent B in solvent A, 1–60% over 25 min, detection at 220 nm.

**Table 2:** Percentages of H–Tyr–Aib–Aib–Phe–Leu–NH_2_ and related deletion peptides obtained in solid-phase assembly

Coupling conditions^a^			Products (%)^b^				
Reagent (4 eq)	Additive (4 eq)	Base (8 eq)	Pentapeptide	Des–Tyr^1^	Des–Aib^2^	Tripeptide	Racemization with Cys(Trt)^c^
HCTU	6-Cl-HOBt	DIEA	20.5	—	79.0	0.5	8.0
HCTU	6-Cl-HOBt	TMP	0.2	2.0	71.3	26.5	1.3
HCTU	6-Cl-HOBt	PS	19.8	—	79.9	0.3	2.0
HCTU	6-Cl-HOBt	DBU	21.7	—	77.4	0.8	2.1
DIC	HOBt	—	2.5	1.7	89.6	6.2	0.1

a The peptide chain was elongated onto an Aib–Phe–Leu Rink amide resin in DMF. The HCTU-mediated couplings were performed by a 1 min preactivation procedure with Fmoc amino acid/HCTU/6-Cl-HOBt (4/4/4 eq) in the presence of the bases (8 equiv) listed in Table 2. The DIC-mediated couplings were performed by a 5 min preactivation procedure with DIC/HOBt (4/4 equiv). The coupling time was 30 min, and concentrations of the active species were 0.20 M.

b Determined by RP-HPLC (220 nm).

c The rate of Cys racemization observed when applying the coupling conditions listed in Table 2 to the synthesis of H–Gly–Cys–Phe–NH_2_ by Fmoc–Cys(Trt)–OH. These data are transcribed from Table 1.

## Examination of the incorporation rate with Fmoc–Cys(Dpm)–OH and Fmoc–Cys(Trt)–OH: synthesis of Boc–Lys(Dnp)–Cys(Dpm/Trt)–Pro–OH

**Figure 2:**
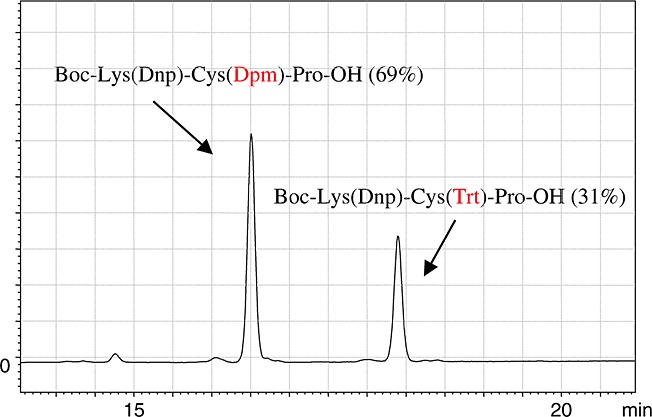
HPLC profile of the products, Boc–Lys(Dnp)–Cys(Dpm)–Pro–OH and Boc–Lys(Dnp)–Cys(Trt)–Pro–OH, obtained by competitive acylation using Fmoc–Cys(Dpm)–OH and Fmoc–Cys(Trt)–OH at the equivalent concentration.

The peptide chain was manually elongated onto H–Pro–Trt(2Cl) resin (0.42 g, 0.24 mmol/g) in DMF. Incorporation of Cys was performed by 1 min preactivation procedure of coupling with Fmoc–Cys(Dpm)–OH + Fmoc–Cys(Trt)–OH/HCTU/6-Cl-HOBt/DIEA (2 + 2/4/4/8 equiv, the concentration of active species, 0.2 M; coupling time, 30 min) in DMF. Fmoc deprotection was carried out with 20% piperidine in DMF (2 × 7 min), followed by washing with DMF (5 × 2 min). After incorporation of Boc–Lys(Dnp)–OH, an aliquot of the resulting peptide resin was treated with hexafluoroisopropanol/CHCl_3_ (v/v, 1/4) at rt for 30 min. The crude product was directly analyzed by HPLC. HPLC conditions: linear gradient of solvent B in solvent A, 30–80% over 25 min, detection at 340 nm (Figure 2).

## Examination of the *C*-terminal Cys racemization during exposure to 20% piperidine/DMF

**Figure 3:**
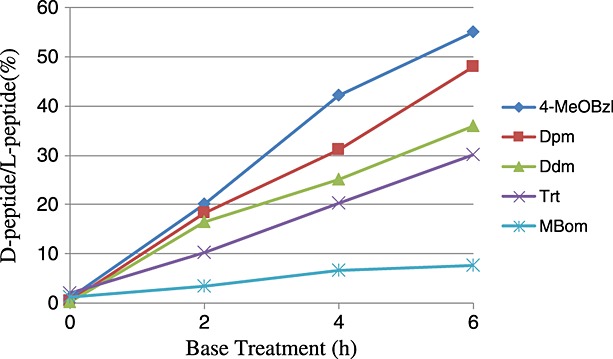
Racemization of the *C*-terminal Cys esterified to Trt resin during exposure of 20% piperidine/DMF. The rate of Cys racemization was defined as (Bz–Ser–d-Cys–OH)/(Bz–Ser–l-Cys–OH) × 100.

The protected resin [Bz–Ser(tBu)–Cys(X)–NovaSyn®TGT resin] (0.1 mmol), where X is Trt, Dpm, 4,4′-dimethoxydiphenylmethyl (Ddm), 4-MeOBzl, or MBom, was treated with 20% piperidine in DMF (5 ml) at room temperature. After 2, 4, 6, and 24 h, an aliquot of each peptide resin was treated with TFA/TIS/H_2_O/PhSH at rt for 1 h. The crude peptides were directly analyzed by HPLC. HPLC conditions: linear gradient of solvent B in solvent A, 1–60% over 25 min, detection at 220 nm (Figure 3).

## Results and Discussion

During incorporation of Fmoc amino acid derivatives onto a growing peptide chain, their racemization is known to more or less occur at the activating and coupling steps with phosphonium or uronium reagents such as PyBOP or HBTU, respectively, in the presence of tertiary amines. In general, racemization of amino acids occurs via oxazolone and direct enolization, and racemization of almost all Fmoc amino acid derivatives proceeds via oxazolone formation, although the extent is inconsequential. In contrast, Cys tends to be readily racemized via enolization because of stabilization of the carbanion formed on *α*-proton abstraction 11. When applying the phosphoniumin/uronium reagents to the SPPS, the addition of tertiary amines is a necessary prerequisite to form the carboxylate ion of the *C*-component to be coupled with the *N*-component. Therefore, the prolongation of preactivation with phosphoniumin/uronium reagents may especially increase the rate of Cys racemization in a time-dependent fashion. To reduce the base-catalyzed Cys racemization during its incorporation to an acceptable level (<1.0%), the carbodiimide-mediated coupling method has been recommended 2. When phosphonium/uronium reagents have to be used to incorporate of the Cys derivatives, alternative procedures are needed: that is, the omission of preactivation and/or the addition of weaker tertiary amines, such as 2,4,6-trimethylpyridine (TMP), compared with the ordinarily used DIEA 2. Thus, we examined the effects of preactivation duration, bases, and solvents used for the coupling reactions on racemization of Cys during its incorporation mediated by phosphonium/uronium reagents.

### Cys racemization and Coupling Efficiency Associated with the Use of Phosphonium/Uronium Reagents

The rate of Cys racemization during its incorporation mediated by HCTU was examined by synthesizing a model peptide designed by Barany *et al*., H–Gly–Cys–Phe–NH_2_, via the conventional SPPS 12. The peptide chain was elongated onto a Rink amide resin using the coupling procedure with Fmoc–Cys(Trt)–OH/HCTU/6-Cl-HOBt/base (4/4/4/8 equiv) in DMF or DCM/DMF, where 1,8-diazabicyclo[5.4.0]undec-7-ene (DBU), DIEA, Proton Sponge (PS), or TMP was used as a base. The effect of the duration of preactivation with HCTU in DMF on Cys racemization is shown in Figure 1, and the effects of bases and solvents on racemization of Cys when employing a 1 min preactivation procedure with HCTU are summarized in Table 1. The racemization rate of Cys increased regardless of the basicity of tertiary amines as the duration of preactivation was prolonged (Figure 1). Note that the rate of Cys racemization could not be suppressed to less than 1.0% during the coupling reaction in DMF even when omitting the preactivation and/or substituting the weaker base TMP for DIEA when employing Cys(Trt). The products prepared using DIEA (p*K*a, 11.4), PS (p*K*a, 12.3), DBU (p*K*a, 12), and TMP (p*K*a, 7.43) were found to contain significant amounts of d-Cys isomers in that order (Table 1). This order in regard to the basicity that was comparable with that observed with the racemization rate of the *C*-terminal residues located on the *C*-components during the segment coupling mediated by phosphonium/uronium reagents 13. Stronger bases than DIEA led to remediation of Cys racemization, rather aggravation of it. This was considered to be due to a rapid coupling in the presence of the stronger bases. Clearly, not only the basicity but also the steric hindrance of bases seem to be strongly involved with the abstraction of the *α*-hydrogen atom from the activated *C*-component or the derived oxazolone. As for Cys racemization when using the Acm group, the results obtained by synthesizing the same model peptide using Fmoc–Cys(Acm)–OH were juxtaposed with those of Fmoc–Cys(Trt)–OH as a function of the coupling conditions in Table 1. The orthogonal protection combination of the Acm and Trt groups for Cys has been frequently used for the regioselective formation of disulfide bonds. The rate of racemization with the Acm group was generally fairly low when compared with that of the Trt group. In particular, substitution of PS or DBU for DIEA afforded an acceptable level of racemization with Cys(Acm) (<1.0%) even in the case of HCTU-mediated coupling performed in DMF, while no measures were taken to avoid racemization with Fmoc–Cys(Trt)–OH. On the other hand, lowering the polarity of the coupling solvent, for example, dilution of DMF with DCM, was found to reduce racemization of Cys to some extent, as had been previously reported 2,12. However, the synthetic procedure would be complicated if the protocols for coupling solvents had to be changed every time the incorporation of Cys is performed, especially in the case of machine-assisted SPPS. Furthermore, in terms of the coupling efficiency, the recommended method to reduce Cys racemization, that is, the DIC/HOBt or HCTU/6-Cl-HOBt/TMP method, was found to be significantly inferior to the standard HCTU-mediated coupling method (HCTU/6-Cl-HOBt/DIEA) as shown in Table 2. The coupling efficiencies arising from the respective coupling conditions were estimated by synthesizing an enkephalin analog, H–Tyr–Aib–Aib–Phe–LeuNH_2_, with the guidance of its yield and product profile 14.

### Acid-labile *S*-protecting Groups Capable of Reducing Cys Racemization

We tried to find an acid-labile *S*-protecting group on Cys that could efficiently suppress the rate of Cys racemization to an acceptable level (<1.0%) even when incorporation of its Fmoc derivative is carried out under general-use SPPS coupling conditions, in which a 1 min preactivation procedure of coupling with Fmoc amino acid/HCTU/6-Cl-HOBt/DIEA (4/4/4/8 equiv) is performed in DMF or 1-methyl-2-pyrrolidinone. These coupling conditions secure a dynamic of coupling efficiency with HCTU as is clear from the results in Table 2. The base-catalyzed racemization of Cys is considered to proceed via enolization because of stabilization of the carbanion formed on *α*-proton abstraction. To destabilize this enol form, an *S*-protecting group that can possess an electron donating effect would be essential to prevent Cys racemization. This effect was clearly interpreted by a correlation observed between racemization propensities of Cys and electronic properties of the Bzl-type protecting groups on Cys as shown in Table 3. Although the phenyl group intrinsically possesses a weak electron donating ability, a substituent on the aromatic ring greatly affects the features of the entire Bzl-type protecting group. Thus, increasing the electron donating effect of the substituent on the Bzl-type protecting groups leads to a decrease in the rate of Cys racemization. In this context, we recently designed the MBom group as an *S*-protecting group having an electron donating property (Table 4) 10. The MBom group was found to be accompanied by an acceptable level of racemization of Cys (0.4%) during its incorporation mediated by phosphonium/uronium reagents in conventional SPPS. Even in the case of the MW-assisted SPPS performed at 50/80 °C, Fmoc–Cys(MBom)–OH caused a significant reduction in the level of racemization (0.8/1.3%), while Fmoc Cys(Trt)–OH led to a considerable level of racemization (10.9/26.6). Besides electron donating properties, a sterically hindered protecting group for Cys capable of shielding the sulfur atom could be expected to destabilize the enol form involved in Cys racemization. The diphenylmethyl (Dpm) group 15 was found to effectively reduce Cys racemization (1.2%) compared with the Bzl group (5.3%). In addition, *p*-substitution of the methoxy group at the Dpm group, that is, Ddm group 15, caused further reduction in the level of racemization (0.8%). However, the most sterically hindered Trt group was inferior for suppressing racemization compared to the less sterically hindered Dpm group, although both protecting groups are considered to not differ much in their electronic characteristics. This discrepancy could be attributable to a slow coupling process of Fmoc–Cys(Trt)–OH by its very nature of steric hindrance, resulting in promoting Cys racemization 16. Competitive acylation using Fmoc–Cys(Dpm)–OH and Fmoc–Cys(Trt)–OH at equivalent concentrations onto a H–Pro–Trt(2-Cl) resin showed that the former could be preferentially incorporated and was accompanied by less racemization than the latter (Figure 2).

**Table 3:** Racemization propensities for the Bzl-type protecting groups on Cys^a^

Protecting group	Racemization (%)^b^
4-NO_2_Bzl	8.8
Bzl	5.3
4-MeOBzl	1.7
Tmob^c^	0.6

a The rate of Cys racemization was determined by synthesizing the model peptide, H–Gly–Cys–Phe–NH_2_. The peptide chain was elongated onto a Rink amide resin using a 1 min preactivation procedure of coupling with Fmoc amino acid/HCTU/6-Cl-HOBt/DIEA (4/4/4/8 equiv) in DMF. The coupling time was 30 min, and concentrations of the active species were 0.20 M.

b Defined as (H–Gly–d-Cys–Phe–NH_2_)/(H–Gly–l-Cys–Phe–NH_2_) × 100.

c Tmob: 2,4,6-trimethoxybenzl.

**Table 4:** Racemization of Cys during synthesis of the model peptide, H–Gly–Cys–Phe–NH_2_, as a function of the Cys protecting group

	Racemization (%)^a^			
Conditions	Trt	MBom	Dpm	Ddm
Conventional SPPS^b^	8.0	0.4	1.2	0.8
MW-assisted SPPS^c^				
50 °C	10.9	0.8	3.0	1.8
80 °C	26.6	1.3	4.5	2.5

a Defined as (H–Gly–d-Cys–Phe–NH_2_)/(H–Gly–l-Cys–Phe–NH_2_) × 100.

b The HCTU-mediated reactions were performed using a 1 min preactivation procedure of coupling with Fmoc amino acid/HCTU/6-Cl-HOBt/DIEA (4/4/4/8 equiv) in DMF. The coupling time was 30 min, and concentrations of the active species were 0.20 M.

c MW heating was performed using the same coupling procedure as the conventional SPPS in a 25 ml polypropylene open vessel placed into the MW cavity of a 300 W single-mode manual MW peptide synthesizer (CEM, Discover SPS) set at 50 or 80 °C; power pulsing sequences of 30 W were used for Cys coupling steps (5 min).

### Racemization of the *C*-terminal Cys Esterified to the Solid Support

Racemization of the *C*-terminal Cys esterified to resins also occurs during repetitive *N^α^*-Fmoc deprotection using piperidine 17. Even when employing a Trt-type resin with the aid of steric hindrance, racemization of the *C*-terminal Cys associated with the base treatment is likely to occur. To estimate the racemization rate arising during the repetitive Fmoc deprotection reactions, the Bz–Ser(*t*Bu)–Cys(X)–NovaSynTGT resin, where X is MeOBzl, Dpm, Ddm, Trt, or MBom, was exposed to 20% piperidine/DMF for a given period Increasing the steric hindrance from the Bzl-type to Dpm-type and Trt-type groups led to a decrease in the racemization rate of the *C*-terminal Cys as shown in Figure 3. Furthermore, substitution of the methoxy groups at the aromatic rings of the Dpm group was found to lead to an obvious lowering in its racemization. In concert with the effect of steric hindrance, the electron donating properties also caused a significant reduction in the rate of racemization in the same manner observed in the HCTU-mediated coupling of the Cys derivatives. When Cys(MBom) was used, a lower rate of racemization (6.4%) was detected even after a 6 h treatment with 20% piperidine/DMF. However, this duration of the base treatment is estimated to correspond with that needed for synthesizing 20–30 amino acid peptides in the conventional SPPS. Therefore, more attention should be paid to the suppression of this racemization with the *C*-terminal Cys linked to a hydroxyl resin, especially when synthesizing a long peptide.

## Conclusion

Racemization of Cys is inevitable during its incorporation if phosphonium/uronium salt-based reagents are employed in the presence of tertiary amines in DMF or 1-methyl-2-pyrrolidinone. In particular, addition of the ordinarily used DIEA leads to a considerable rate of Cys racemization. Substitution of PS or DBU for DIEA in the coupling procedure using phosphonium/uronium reagents was found to be essential not only for reducing the rate of Cys racemization but also for securing their intrinsic dynamic of coupling efficiency and effectiveness. Thus, this procedure using PS or DBU caused a significant reduction in the level of racemization with Cys(Acm) to an acceptable level (<1.0%) while that with Cys(Trt) was not sufficiently suppressed. The Ddm and MBom groups that are acid-labile *S*-protecting groups comparable with the Trt group were demonstrated to efficiently prevent racemization during the Cys incorporation compared with the Trt group even when performing the conventional and MW-assisted SPPS mediated by phosphonium/uranium reagents in the presence of DIEA.

## Abbreviations

Acm: acetamidomethyl
Boc: *t*-butoxycarbonyl
*t*Bu: *t*-butyl
Bum: *t*-butyloxymethyl
Bzl: benzyl
6-Cl-HOBt: 6-chloro-1-hydroxybenzotriazole
DBU: 1,8-diazabicyclo[5.4.0]undec-7-ene
Ddm: 4,4′-dimethoxydiphenylmethyl
Dpm: diphenylmethyl
DIC: *N*,*N*′-diisopropylcarbodiimide
DCM: dichloromethane
DIEA: *N*,*N*-diisopropylethylamine
DMF: *N*,*N*-dimethylformamide
DNP: 2,4-dinitrophenyl
EDC · HCl: 1-ethyl-3-(3-dimethylaminopropyl)carbodiimide hydrochloride
ESI-MS: electrospray ionization mass spectrometry
Fmoc: 9-fluorenylmethyoxycarbonyl
HBTU: 1-[*bis*(dimethylamino)methylene]-1*H*-benzotriazolium 3-oxide hexafluorophosphate
HCTU: 1-[*bis*(dimethylamino)methylene]-5-chloro-1*H*-benzotriazolium 3-oxide hexafluorophosphate
HOBt: 1-hydroxybenzotriazole
HPLC: high-performance liquid chromatography
MBom: 4-methoxybenzyloxymethyl
MW: microwave
NCL: native chemical ligation
NMP: 1-methyl-2-pyrrolidinone
PS: Proton Sponge
PyBOP: 1*H*-benzotriazol-1-yloxytris(pyrrolidino)phosphonium hexafluorophosphate
RP-HPLC: reversed phase HPLC
SAL: supper acid labile
SPPS: solid-phase peptide synthesis
TFA: trifluoroacetic acid
TIS: triisopropylsilane
TMP: 2,4,6-trimethylpyridine
Trt: trityl
Trt(2-Cl): 2-chlorotrityl.
